# scGIR: deciphering cellular heterogeneity via gene ranking in single-cell weighted gene correlation networks

**DOI:** 10.1093/bib/bbae091

**Published:** 2024-03-14

**Authors:** Fei Xu, Huan Hu, Hai Lin, Jun Lu, Feng Cheng, Jiqian Zhang, Xiang Li, Jianwei Shuai

**Affiliations:** Department of Physics, Anhui Normal University, Wuhu 241002, China; Wenzhou Institute and Wenzhou Key Laboratory of Biophysics, University of Chinese Academy of Sciences, Wenzhou 325001, China; Institute of Applied Genomics, Fuzhou University, Fuzhou 350108, China; Wenzhou Institute and Wenzhou Key Laboratory of Biophysics, University of Chinese Academy of Sciences, Wenzhou 325001, China; Department of Physics, Anhui Normal University, Wuhu 241002, China; School of Medical Imageology, Wannan Medical College, Wuhu 241002, China; Department of Physics, and Fujian Provincial Key Lab for Soft Functional Materials Research, Xiamen University, Xiamen 361005, China; Department of Physics, Anhui Normal University, Wuhu 241002, China; Department of Physics, and Fujian Provincial Key Lab for Soft Functional Materials Research, Xiamen University, Xiamen 361005, China; Wenzhou Institute and Wenzhou Key Laboratory of Biophysics, University of Chinese Academy of Sciences, Wenzhou 325001, China; Oujiang Laboratory (Zhejiang Lab for Regenerative Medicine, Vision and Brain Health), Wenzhou 325001, China

**Keywords:** single-cell RNA sequencing, heterogeneity analysis, gene expression weighted network, gene correlation network, gene importance

## Abstract

Single-cell RNA sequencing (scRNA-seq) has emerged as a powerful tool for investigating cellular heterogeneity through high-throughput analysis of individual cells. Nevertheless, challenges arise from prevalent sequencing dropout events and noise effects, impacting subsequent analyses. Here, we introduce a novel algorithm, Single-cell Gene Importance Ranking (scGIR), which utilizes a single-cell gene correlation network to evaluate gene importance. The algorithm transforms single-cell sequencing data into a robust gene correlation network through statistical independence, with correlation edges weighted by gene expression levels. We then constructed a random walk model on the resulting weighted gene correlation network to rank the importance of genes. Our analysis of gene importance using PageRank algorithm across nine authentic scRNA-seq datasets indicates that scGIR can effectively surmount technical noise, enabling the identification of cell types and inference of developmental trajectories. We demonstrated that the edges of gene correlation, weighted by expression, play a critical role in enhancing the algorithm’s performance. Our findings emphasize that scGIR outperforms in enhancing the clustering of cell subtypes, reverse identifying differentially expressed marker genes, and uncovering genes with potential differential importance. Overall, we proposed a promising method capable of extracting more information from single-cell RNA sequencing datasets, potentially shedding new lights on cellular processes and disease mechanisms.

## INTRODUCTION

Single-cell RNA sequencing (scRNA-seq) is a technology that measures RNA transcripts at the single-cell level, providing technical support for a more in-depth study of the heterogeneity and complexity among individual cells [1–3]. scRNA-seq technology has revealed the composition of different cell types and functions in highly organized tissues, organs, or organisms. This advancement promotes research on complex diseases, development, and evolution, offering broad prospects for maintaining human health [4, 5]. Developing effective analytical tools to extract information from scRNA-seq data can provide a high-resolution perspective on intercellular differences, including accurate identification of cell types, recognition of various expression patterns, and the discovery of potential biological mechanisms. These tools aid in gaining a better understanding of the function, specificity, and interactions of cells [6]. However, the sparse and noisy nature of single-cell sequencing data poses considerable challenges in extracting reliable and meaningful information. Currently, developing new tools to process single-cell sequencing data from a multidisciplinary perspective is a research focus in bioinformatics [7–9].

Traditional analysis tools that are based on single-cell gene expression matrix (GEM) have yielded fruitful results in downstream analyses, such as cell clustering analysis and developmental potential inference [10–13]. However, these tools have neglected the instability of raw expression data and the correlation between genes. The biological system is a highly complex non-linear system, and noise from both internal and external sources can lead to significant variations in gene expression in individual cells [14]. These variations might even result in cells executing entirely different fate decisions [15]. There is significant evidence that gene expression within cells is controlled by a precise signal transduction network [16]. Despite significant differences in gene expression among cells of the same type, their gene correlation networks are relatively stable [17]. Inferring gene correlation networks from gene expression data is an important research topic in the analysis of single-cell sequencing data [18–20]. Recent studies show that the network information within single-cell-specific gene correlation networks is effective for analyzing cell heterogeneity and complexity [17, 21]. However, relying solely on network information analysis from gene correlation networks may lead to the loss of some expression information. Therefore, there is an urgent need for a new analysis tool that simultaneously considers both gene network information and expression information.

In this study, we proposed a single-cell sequencing data analysis tool, Single Cell Gene Importance Ranking (scGIR), based on complex network theory and page ranking algorithms. The model, constructed on complex network theory, uniquely integrates gene expression information with gene correlation network data, creating a single-cell weighted gene correlation network for the first time. The scGIR model comprises three main modules: the gene correlation network construction module, the gene expression weighted module, and the importance ranking matrix construction module. The gene correlation network construction module infers the correlation between genes at the single-cell level. The gene expression weighted module incorporates the expression information of gene nodes on the single-cell-specific gene correlation network and converts them into edge weights. The importance matrix construction module establishes a random walk model on the single-cell weighted gene correlation networks. Drawing inspiration from the classic web page ranking algorithm, this module introduces a weighted PageRank for the single-cell weighted gene correlation network [22]. scGIR effectively converts the single-cell GEM into a single-cell gene importance matrix (GIM), and the sizes of these two matrices are consistent. Finally, we illustrated the advantages of using GIM in the analysis of single-cell heterogeneity analysis. Computationally, GIM maintains a similar scale to GEM and does not introduce increased computational complexity. Leveraging the scGIR methodology enables a more refined examination of cellular subtypes, elucidating differences in gene correlation networks among these subtypes. Furthermore, scGIR has the capability to identify key genes with insignificantly differential expression at the network level, revealing richer systemic biological information within scRNA-seq datasets. Overall, scGIR proves to be an effective tool for accurately identifying cellular heterogeneity from vast amounts of single-cell sequencing data, contributing to a better understanding of the diversity of life phenomena and the intricacies of cell development and evolution.

## MATERIALS AND METHODS

### Datasets

In this study, we compiled nine scRNA-seq datasets from diverse species and tissues, gathering data from previous research projects and the National Center for Biotechnology Information database (Table 1) The *PBMC4k* dataset of peripheral blood mononuclear cells comprises 4340 cells, including eight distinct cell types. The *Mouse Bladder Cells* dataset is sourced from the Mouse Cell Atlas, providing comprehensive gene expression data for nearly 50 distinct types of mouse cells, covering over 0.4 million individual cells [23]. We selected a subset of 2746 cells from mouse bladder tissue for heterogeneity analysis. The *Li* dataset results from scRNA-seq performed on 11 primary colorectal tumors and their corresponding normal mucosa, offering valuable insights into the transcriptional heterogeneity in colorectal tumors and their microenvironment [24]. The *Chu-time* dataset encompasses 758 cells sampled at six distinct time points throughout the process of cellular differentiation, from human embryonic stem cells into qualitative endoderm cells [25]. The *Gokce* dataset comprises 705 cells of 10 different types, including immune cells, astrocytes, vascular cells, and neuron cells [26]. The *Chu-type* dataset is derived from lineage-specific progenitor cells obtained from human embryonic stem cells, consisting of 1018 cells belonging to seven different cell types [25]. The HND dataset comprises 483 cells at six different time points from human neuronal differentiation experiment [27]. The Tasic dataset comprises 49 transcriptionally cell types from the primary visual cortex of adult mice, covering 23 GABAergic, 19 glutamatergic and 7 non-neuronal cell types. We selected only the GABAergic group for subsequent analyses [28]. The *Trapnell* dataset is obtained from primary adult myocytes and comprises 372 cells belonging to four different cell types [29].

**Table 1:** Nine scRNA-seq datasets are used in this paper

Datasets	Number of Cells	Number of Genes	Cell Types	Data Sources
PBMC4k	4340	16 653	8	10X genomics
Mouse bladder cells	2746	20 670	16	figshare.com
Li	359	57 241	5	GSE81861
Chu-time	1018	8700	7	GSE75748
Gokce	705	18 840	10	GSE82187
Chu-type	758	9600	6	GSE75748
HND	483	8800	6	GSE102066
Tasic	1634	24 057	23	GSE71585
Trapnell	372	6600	4	GSE52529

### Data preprocessing

scGIR is motivated by the fact that the reaction rate of genes or proteins molecular interactions in intracellular biochemical reactions are positively correlated with molecular concentration [30–33]. We proposed to weight the gene–gene correlation in scRNA-seq data based on gene expression levels and evaluate the importance of genes within single cells using the PageRank algorithm [22]. To construct a reliable single-cell gene correlation network, we must overcome the challenges posed by sparse and noisy single-cell sequencing data. Therefore, we preprocessed the scRNA-seq data by removing genes that are expressed in only a very small number of cells, and cells with abnormally low or high total gene expression levels [34]. We also performed a logarithmic transformation to the original gene expression data (*E_orig._*) in the sequencing data to reduce the degree of dispersion, as follows:


(1) \begin{equation*} E=\mathit{\log}\left({E}_{orig.}+1\right) \end{equation*}

**Figure 1:**
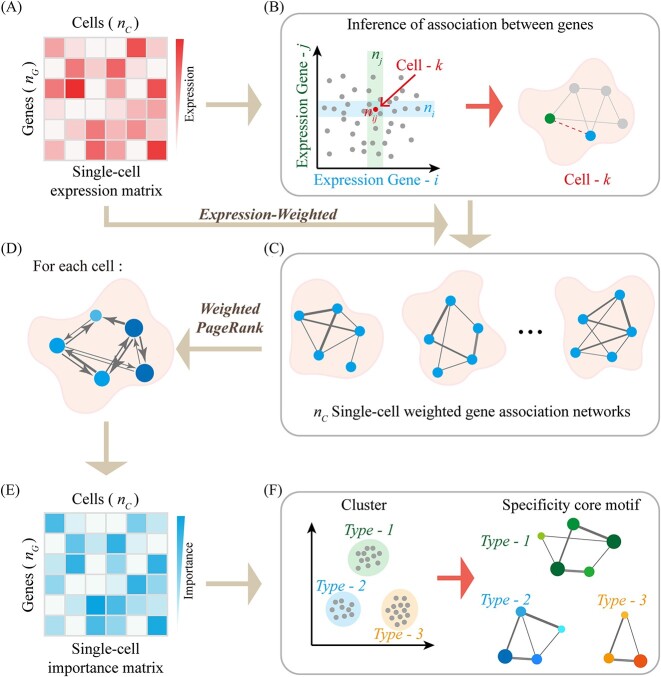
Schematic diagram of scGIR workflow. (**A**) The preprocessed single-cell GEM. (**B**) All cells were mapped onto a two-dimensional gene expression plane, and statistical independence was used to determine whether there was a correlation between any two genes across all cells. (**C**) Edges in the single-cell gene correlation network were weighted by gene expression levels. (**D**) Construct random walk models on each of the weighted gene correlation networks for all single cells. (**E**) A single-cell GIM was obtained through a ranking algorithm. (**F**) Cellular heterogeneity was examined, and specific core gene correlation motifs for each cell type were inferred by analyzing the single-cell GIM.

To optimize the computational cost of constructing single-cell gene correlation networks and downstream analysis, we used feature extraction techniques to perform dimensionality reduction on the data. In this study, we selected the top 2000 highly variable genes from all scRNA-seq data and represented the data as a GEM (Figure 1A).

### Construction of single-cell gene correlation network

Building upon the statistical independence assumption between two genes, the identification of correlation between gene pairs from the GIM and the construction of a single-cell gene correlation network have been subjects of prior research. [17]. As shown in Figure 1B, two genes *i* and *j* are arbitrarily selected from GIM, and all cells are mapped to the two-dimensional gene space. In cell *k*, the independence index of gene *i* and gene *j* is given by the following formula:


(2) \begin{equation*} {\rho}_{ij}^k=\frac{n_{ij}^k}{n_C}-\frac{n_i^k}{n_C}\cdot \frac{n_j^k}{n_C} \end{equation*}

where *n_i_^k^* and *n_j_^k^* denote the number of cells in which the expression levels of gene *i* and gene *j* are close to that of cell *k*. *n_ij_^k^* represents the number of cells in which the expression levels of both gene *i* and gene *j* are close to that of cell *k*, indicating the intersection of *n_i_^k^* and *n_j_^k^*. *n_C_* represents the total number of cells in GIM. The range of independence index *ρ_ij_^k^* is −1 to 1. For simplicity, we fixed *n_i_^k^* and *n_j_^k^* at 0.1*n_C_* for all cells. When genes *i* and *j* are independent across all cells, it has been demonstrated that *ρ_ij_* follows an approximately standard normal distribution. The significance level, set consistently at 0.01 as the threshold, serves as the criterion for assessing the correlation between two genes within individual cells, aligning with previous relevant reports [17]. Cells falling within the rejection interval are deemed to lack correlation between the two genes, whereas cells within the acceptance interval are considered associated. By applying this criterion, we could extract *n_C_* single-cell gene correlation networks from the single-cell GEM.

### Gene expression weighted correlation

The presence of a correlation between two genes suggests a potential for mutual regulation, and the expression levels of the genes are positively correlated with the weight of their correlation. The correlation weight of gene *i* to gene *j* in cell *k* is defined as follows:


(3) \begin{equation*} {W}_{ij}^k=\frac{E_i^k}{\sum \limits_{m\in{L}_j^k}{E}_m^k} \end{equation*}

where *E_i_^k^* represents the expression level of gene *i* in cell *k*. *L_j_^k^* represents the set of adjacent genes of gene *j* in the gene correlation network in cell *k*, and *E_m_^k^* represents the expression level of gene *j* adjacent genes. The operation of expression-weighted transformed the single-cell gene correlation networks into single-cell weighted gene correlation networks (Figure 1C). It is imperative to clarify that our approach exclusively relies on pre-processed sequencing data to construct a single-cell weighted gene correlation network, employing an unsupervised methodology.

### Ranking of gene importance

PageRank algorithm has been widely employed to assess the significance of web pages on the Internet [35]. The algorithm relies on two fundamental assumptions: (i) the quantity assumption, which posits that a web page’s importance increases with the number of inbound links it receives from other web pages and (ii) the quality assumption, which suggests that when multiple high-quality web pages link to a particular web page, it signifies its importance. We employed PageRank algorithm to assess the importance of genes within single cells. In single-cell biology, the gene correlation network can be equivalently likened to the Internet, with each gene functioning like a web page. The foundational assumptions of the PageRank algorithm are reasonably valid in single-cell analysis. Specifically, regulated by numerous others, are crucial, and genes connected to them are likely important as well. Assessing gene importance in single cells via the PageRank algorithm involves constructing a random walk model on the gene correlation network. In the process of random walks, transitioning from the current gene node to the next gene node occurs randomly based on the correlative relationships within the network, unaffected by the previously traversed pathways. This aligns with the characteristics of a Markov process. Assuming equal probability for transitions between associated genes, the PageRank score is calculated using the following equation:


(4) \begin{equation*} P{R}_i^k=\left(1-d\right)+d\sum \limits_{j\in{L}_i^k}\frac{P{R}_j^k}{N_j^k} \end{equation*}

where *PR_i_^k^* represents the PageRank score of gene *i* in cell *k*. *d* is the dampening factor and is fixed to 0.85 [36]. *L_i_^k^* represents the set of neighbor-associated genes for gene *i* in cell *k*, and *N_j_^k^* denotes the number of genes in cell *k* that are associated with the presence of gene *j*. In this study, we constructed a random walk model for each single-cell gene’s weighted correlation network, and iteratively calculated the access probability of each gene node from the gene correlation network and its weights (Figure 1D). Unlike the conventional approach of assigning PageRank values based on node in-degree and out-degree, our approach considered the correlation weight for assigning PageRank values, detailed as follows:


(5) \begin{equation*} P{R}_i^k=\left(1-d\right)+d\sum \limits_{j\in{L}_i^k}P{R}_j^k\cdot{W}_{ji}^k. \end{equation*}

Employing a single-cell data-based weighted gene correlation network, we determined the importance index of each gene. This approach enabled us to develop a single-cell GIM, maintaining the original dimensions of the single-cell GEM (Figure 1E). Utilizing a scRNA-seq data analysis algorithm, we used the GIM to reduce dimensionality, cluster cells for heterogeneity analysis, and discern core gene motif variations among different cell types (Figure 1F).

## RESULTS

### scGIR significantly improves cell type identification

**Figure 2:**
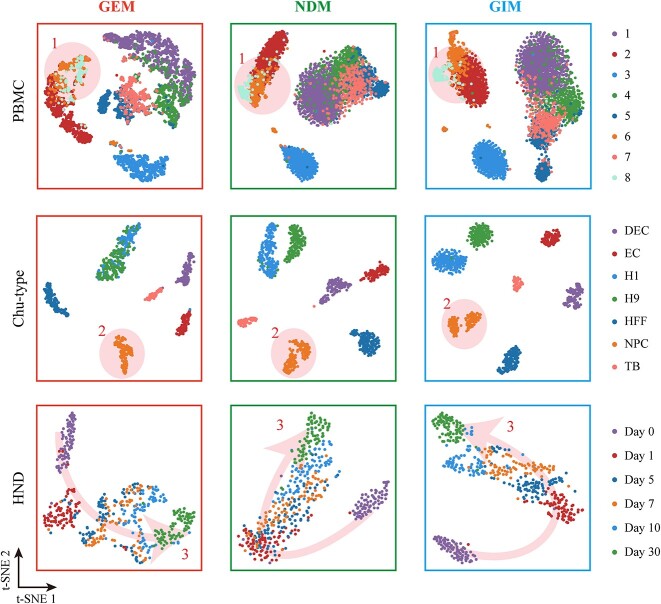
Comparison of GEM (left column), NDM (middle column), and GIM (right column) on non-linear dimensionality reduction (t-SNE) visualization for three typical datasets. Shadow-1 shows GIM’s improved ability to distinguish cell types in PBMC data. Shadow-2 shows improved identification of NPC subtypes in the Chu-type dataset. Arrow-3 highlights GIM’s improved inference of cell differentiation trajectories in the HND dataset.

To validate the effectiveness of scGIR in analyzing scRNA-seq data, we performed non-linear dimensionality reduction (t-SNE) visualization on the GEM, gene correlation network degree matrix (NDM), and GIM of the nine datasets outlined in Table 1 [37]. The visualization results of the *PBMC4k*, *Chu-type* and *Wang* datasets are shown in Figure 2. Comparing the dimensionality reduction visualization results of the three matrices, we observed superior performance of the GIM generated by scGIR, which integrates both expression information and gene correlation network data. As shown in Figure 2, the GIM distinctly highlights the position of the eighth cell type in the *PBMC4k* dataset, providing a clearer separation of Shadow 1. After dimensionality reduction of the *Chu-type* dataset, GIM not only accurately identified different cell types but also separated different cell subtypes within the neural progenitor cell (NPC) cluster. Particularly, scGIR demonstrates significant potential in the realm of generating cell lineage trajectories. In the analysis of the neuronal time-course differentiation dataset (HND), GIM accurately predicted the developmental states and trajectories of neuronal cells, as indicated by arrow 3 in Figure 2. Day 0 cells exhibit heightened differentiation potential, showcasing a significant distinction from cells in the later stages of differentiation. The GIM analysis reveals a distinct spatial separation of day 0 cells within the phase space compared to their differentiated counterparts. And, GIM demonstrates high accuracy in identifying cell types at different differentiation states, and it accurately depicts the temporal continuity of cell state transitions during development. The dimensionality reduction visualizations for the remaining 6 datasets are presented in Figure S1.

To quantify the effectiveness and universality of scGIR in analyzing cell heterogeneity in single-cell sequencing data, we applied seven distinct clustering methods to GEM, NDM, and GIM data across 9 datasets. Using the Adjusted Rand Index (ARI) as a conventional evaluation metric, we compared the clustering results against the original cell type labels [17, 21, 38]. The data presented in Table 2 indicates that GIM outperformed GEM and NDM on the majority datasets and clustering algorithms. These quantitative metrics provide compelling evidence that the scGIR algorithm, by integrating expression information and gene correlation networks, severs as a highly effective and reliable tool for the analysis of cell heterogeneity in scRNA-seq data.

**Table 2:** The comparison of GEM, NDM, and GIM in cluster analysis, with the highest score highlighted, as assessed by ARI

Cluster Methods	Matrix	PBMC	Mouse bladder cells	Li	Chu-time	Gokce	Chu-type	HND	Tasic	Trapnell
k-means	GEM	0.74	0.42	0.10	0.64	0.70	0.75	0.39	0.24	0.14
	NDM	0.66	0.32	0.04	0.53	0.49	0.73	0.25	0.22	0.04
	**GIM**	**0.76**	**0.45**	**0.13**	**0.68**	**0.76**	**0.76**	**0.62**	**0.27**	**0.21**
Hierarchical	GEM	0.56	0.43	0.08	0.56	0.71	0.76	0.43	0.23	0.04
	NDM	0.53	0.34	0.05	0.52	0.60	0.80	0.27	0.23	0.07
	**GIM**	**0.67**	**0.46**	**0.15**	**0.67**	**0.82**	**0.99**	**0.62**	**0.28**	**0.23**
k-means (t-SNE)	GEM	0.48	**0.42**	0.09	0.70	0.45	0.96	0.45	0.24	0.14
	NDM	0.51	0.26	0.03	0.75	0.45	0.98	**0.48**	0.20	0.02
	**GIM**	**0.52**	0.41	**0.11**	**0.73**	**0.43**	**0.99**	0.26	**0.26**	**0.22**
Hierarchical(t-SNE)	GEM	0.45	**0.46**	0.09	0.57	0.45	0.97	0.43	0.27	0.14
	NDM	0.51	0.26	0.03	0.76	0.47	0.98	**0.51**	0.24	0.04
	**GIM**	**0.58**	0.42	**0.12**	**0.83**	**0.48**	**0.99**	0.23	**0.27**	**0.21**
k-medoids	GEM	0.38	0.31	0.09	**0.62**	0.26	0.65	0.39	0.24	0.14
	NDM	0.49	0.29	0.06	0.57	**0.32**	0.45	0.31	0.19	0.10
	**GIM**	**0.50**	**0.44**	**0.15**	0.46	0.25	**0.69**	**0.53**	**0.24**	**0.18**
Spectral	GEM	0.48	0.00	0.03	0.00	0.39	0.00	0.10	0.00	0.03
	NDM	0.63	0.14	0.08	0.48	0.54	**0.77**	0.01	0.00	0.01
	**GIM**	**0.73**	**0.52**	**0.12**	**0.67**	**0.70**	0.72	**0.47**	**0.24**	**0.19**
Seurat	GEM	0.40	0.52	0.03	0.54	0.41	0.88	0.65	0.24	0.13
	NDM	0.45	0.53	0.08	0.59	0.40	0.80	0.64	0.25	0.15
	**GIM**	**0.61**	**0.56**	**0.12**	**0.65**	**0.48**	**0.97**	**0.65**	**0.29**	**0.22**

### Gene expression-weighted is a key factor for improving scGIR performance

**Figure 3:**
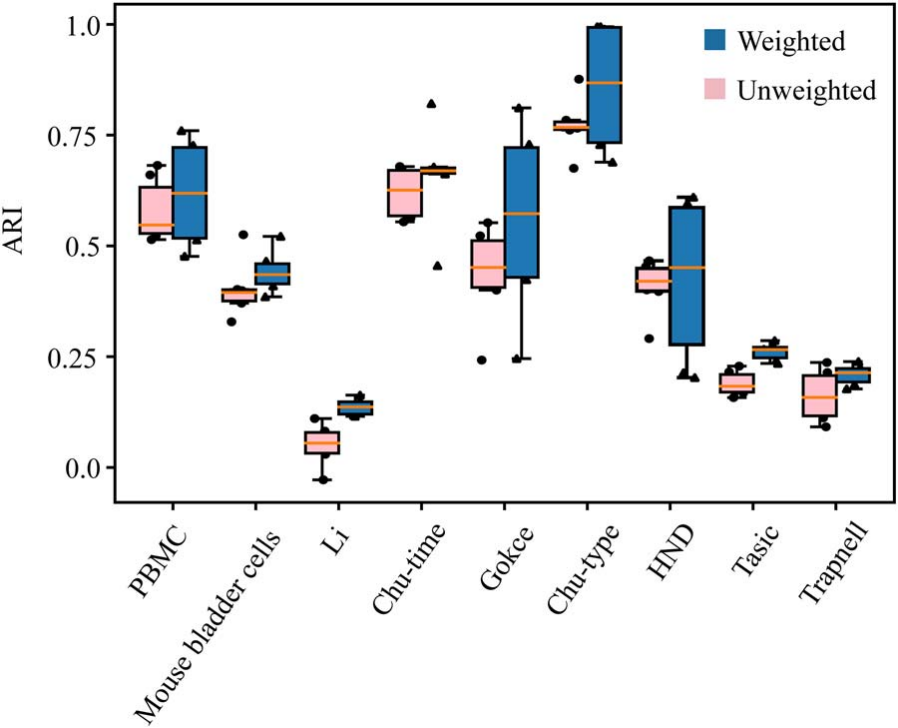
Performance comparison of scGIR methods with and without gene expression-weighted across 9 scRNA-sqe datasets.

To identify the critical role of gene expression-weighted modules, we conducted a comparative analysis between scGIR without gene expression weighting and scGIR incorporating weighted module influences. This comparison involved the application of 6 distinct clustering methods, assessing comprehensive performance across 9 scRNA-seq datasets. To mitigate potential instability in results due to randomness during the random walk process, we conducted 10 repeated measurements for each independent experiment. As depicted in Figure 3, the statistical results depicted in the box plots highlight the contribution of gene expression-weighted modules to enhancing the performance of scGIR to a certain extent. Removing the gene expression-weighted modules resulted in a module importance ranking matrix that connects the single-cell-specific network (CSN), which is an unweighted graph. We used the original PageRank algorithm to calculate node importance instead of the weighted PageRank. The original PageRank algorithm involves a random walk through the directed graph, where the probability of visiting each node converges to a stationary distribution, and the stationary probability value of each node is output as its importance. This importance is not affected by edge weights, and therefore does not allocate different importance based on the weight of each neighboring node’s inbound link. GIM with gene expression-weighted exhibited strong compatibility with various clustering algorithms, showcasing higher average precision compared to its unweighted counterpart.

### Cellular heterogeneity from gene-level and network-level

**Figure 4:**
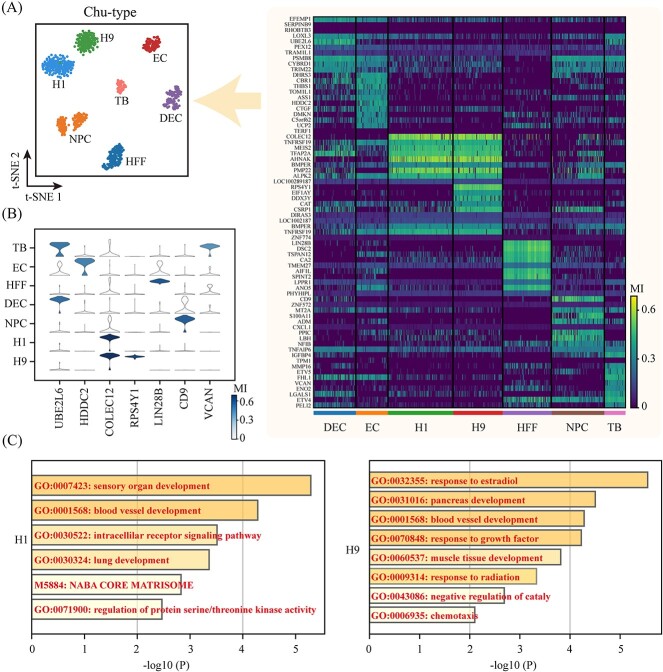
Using the scGIR method to obtain cell heterogeneity and functional enrichment visualization of the GIM analysis of *Chu-type* dataset. (**A**) t-SNE dimensionality reduction visualizes seven cell clusters in the *Chu-type* dataset, and the heatmap shows the mean importance (MI) of the Top-10 marker genes obtained through the Wilcoxon rank-sum test for each cell cluster. (**B**) Visualization of marker genes for each cell cluster. (**C**) Gene Ontology biological processes analysis of the mean importance marker genes in H1 and H9 cell clusters.

The single-cell GIM analysis of cellular heterogeneity is based on the mean importance of gene nodes in the weighted gene correlation networks of individual cell clusters, i.e., the mean importance. Based on average importance for the 7 labeled cell types in the *Chu-type* dataset, we generated a heatmap depicting the top 10 highly variable genes (Figure 4A), employing the Wilcoxon rank-sum test [39]. The t-SNE and *k*-means clustering were employed for visualizing the 7 cell clusters in the *Chu-type* dataset. UMAP and PCA are also extensively used to visualize gene expression data [40, 41]. The results of PCA and UMAP dimensionality reduction visualizations are depicted in Figure S2. In comparison, t-SNE is more suitable for analyzing cellular heterogeneity and discovering novel cell subtypes in the *Chu-type* dataset. We investigated the minimal gene combinations that could distinguish the 7 cell types, and a violin plot was used to display the average importance of the 7 labeled genes in each cell type (Figure 4B). Among these findings, gene UBE2L6 emerged as highly important in both trophoblast-like cells (TB) and definitive endoderm cells (DEC), while gene VCAN showed specificity with elevated importance in TB. VCAN encodes Versican, a protein belonging to the family of proteoglycans, and is a structurally complex macromolecule [42]. Versican plays an important role in trophoblast cells. Notably, genes COLEC12 in H1 human embryonic stem cells (H1) and H9 human embryonic stem cells (H9) displayed high importance, with gene RPS4Y1 showing specificity for high importance exclusively in H9. RPS4Y1, located on the Y chromosome, encodes the protein RPS4Y1, a component of the ribosome, the primary cellular organelle responsible for protein synthesis [43, 44]. As a Ribosomal protein, RPS4Y1 collaborates with others to regulate the protein synthesis process. Furthermore, genes HDDC2, LIN28B and CD9 were specifically identified in endothelial cells (EC), human foreskin fibroblasts (HFF) and neural progenitor cells (NPC), respectively.

To unveil the connections between known biological functions and pathways derived from gene importance, the results of the top 10 highly variable gene importance across seventh cell clusters in the *Chu-type* dataset are depicted in Figures 4C and S3, as revealed through the Matespace Enrichment Analysis [45]. The most important enrichment pathways for the gene importance in H1 cells are associated with sensory organ development and blood vessel development. While the genes’ importance in H9 cells is related to the response to estradiol, pancreas development, blood vessel development, and growth factor response.

**Figure 5:**
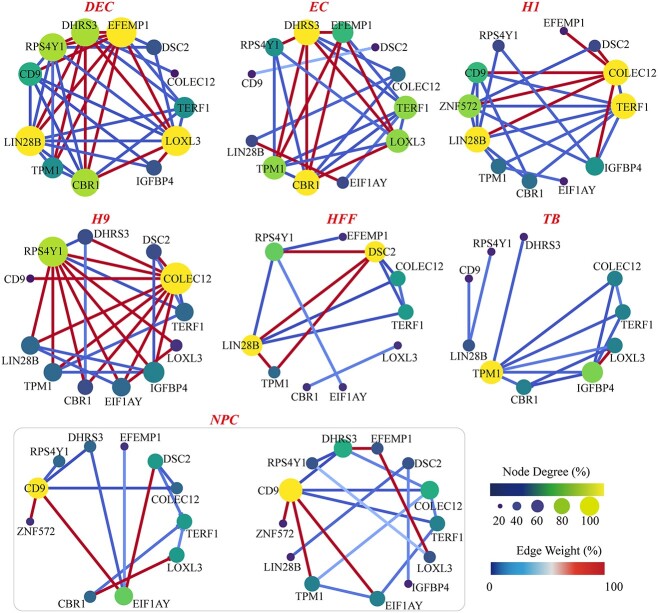
Network analysis of marker genes for seven cell clusters in the *Chu-type* dataset, showing the gene correlation networks of different subtypes of NPC cells. The size of the nodes represents the degree, and the color of the edges represents the expression weight.

The scGIR not only reveals differences in gene expression among different cell clusters but also identifies discrepancies in core gene network motifs among these clusters. Figure 5 shows a weighted correlation network of 14 marker genes across seven cell types in the *Chu-type* dataset, where the color and size of the nodes represent the degree of the nodes, and the color of the edges represents the correlation strength. The weighted marker gene correlation networks for DEC, H1 and H9 clusters exhibit greater complexity, while those for HFF and TB clusters are comparatively sparse. On one hand, cells with high differentiation potential appear to possess a more intricate gene regulatory network, as they necessitate the regulation of various genes at different developmental stages and under diverse environmental conditions to differentiate into various cell types. On the other hand, the complexity of a cell’s gene regulatory network also depends on the functions it needs to execute. The complexity of the weighted gene correlation networks in EC cluster surpasses that of NPC cluster. EC constitute the primary components of blood vessels and lymphatic vessels, playing crucial roles in various physiological processes such as blood clotting, immune responses, and nutrient exchange. The gene correlation network of EC must be capable of responding to diverse physiological and pathological conditions to maintain the normal functionality of blood vessels [46]. Genes with high importance indices in each cell have a larger node degree and higher correlation weights with other genes (highlighted in yellow). Additionally, subtle variations in the weighted network of marked genes are underscored among different subtypes of NPC. In a holistic perspective, the network of subtype 1 appears to be sparser compared to subtype 2. When comparing individual gene nodes, apart from CD9, there are more neighboring genes around the E1F1AY in subtype 1, whereas in subtype 2, the COLEC2 exhibits a higher number of surrounding neighbor genes.

### Reverse identification of expression marker genes by GIM

**Figure 6:**
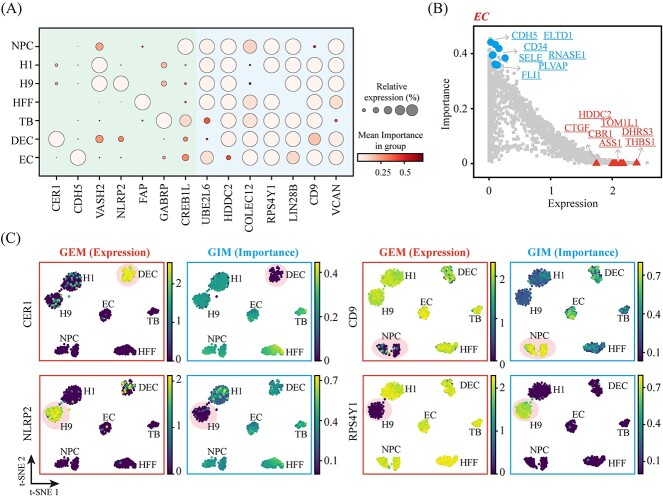
Comparative analysis of expression marker genes and importance marker genes. (**A**) Visualization of 7 expression marker genes and 7 important marker genes. The size of the nodes represents the average expression level, and the color represents the average importance. (**B**) All genes in the EC cell cluster mapped onto a two-dimensional space based on expression and importance, with marker genes highlighted separately. (**C**) Performance of genes CER1, NLRP2, CD9 and RPS4Y1 in the t-SNE plot, with red and blue boxes colored by expression and importance, respectively.

To reconcile the inconsistencies observed between the results of GEM and GIM analyses, we first identified marker genes with differential expression across distinct cell types employing GEM analysis, as presented in Figures S4 and S5. Subsequently, we selected the top highly variable gene from each cluster along with highly variable genes of significant importance from the previous section, as illustrated in Figure 6A. In the bubble plot, the average gene expression level is represented by the size of each point, while the average gene importance is indicated by the color. The outcomes revealed a negative correlation between expression level and importance metrics of marker genes obtained from both GIM and GEM analyses. Specifically, genes with higher expression levels exhibited lower importance, and vice versa.

We projected the average expression level and average importance of all cell clusters onto a two-dimensional space, depicted in Figure 6B and S6. In Figure 6B, importance marker genes in EC cell clusters were highlighted with blue dots, whereas expression marker genes were represented by red triangle dots. It is evident that importance marker genes are primarily concentrated in the upper left corner, while expression marker genes are predominantly located in the lower right corner. CER1 and NLRP2 genes serve as marker genes for the expression levels of DEC and H9 cells, respectively. They are highly expressed in a specific manner within their respective cell clusters, but also exhibit low specificity and importance within these clusters. Conversely, CD9 and RPS4Y1 genes act as importance marker genes for NPC and H9 cells, respectively. They are highly important and specific within their respective cell clusters, but also demonstrate low specificity and expression within these clusters. Overall, GIM not only identifies genes with high differences in importance within gene correlation networks but also serves as a tool to recognize marker genes for differential expression.

### GIM outperforms NDM in identifying ‘dark’ genes with higher precision

If a gene cannot be distinguished as cell-specific based on its expression level but can be distinguished as cell-specific based on other indicators, it is referred to as a ‘Dark’ gene [17]. Here, we report the identification of two ‘Dark’ genes, SERPINB9 and TERF1, using GEM, as depicted in Figure 6A. The expression levels, degree centrality, and gene importance of SERPINB9 and TERF1 were individually mapped onto the dimensionality reduction plot. While the expression of SERPINB9 and TERF1 is relatively pronounced in DEC and H1/9 clusters, their expression in other cell clusters should not be overlooked. The quantitative analysis of gene expression alone fails to elucidate the specific roles played by SERPINB9 and TERF1 in DEC and H1/9 clusters. HDM identified the degree centrality of SERPINB9 and TERF1 as nodes in the single-cell gene correlation networks. However, SERPINB9 exhibited significant degree centrality in both DEC and EC, while TERF1 showed significant degree centrality in H1/9, DEC, EC, HFF, and TB. HDM was also unable to ascertain the specific roles of SERPINB9 and TERF1 in the DEC and H1/9 clusters. GIM, through assessing the gene importance of SERPINB9 and TERF1, reveals heightened specificity of SERPINB9 in DEC compared to other cell clusters. Additionally, TERF1 exhibits increased specificity in H1/9 when compared to other clusters. The protein encoded by SERPINB9 is a serine protease inhibitor, also known as Proteinase Inhibitor 9. On the other hand, the TERF1 gene encodes a protein known as telomere-binding protein, which serves as an inhibitor of telomerase throughout all stages of the cell cycle [47].

**Figure 7:**
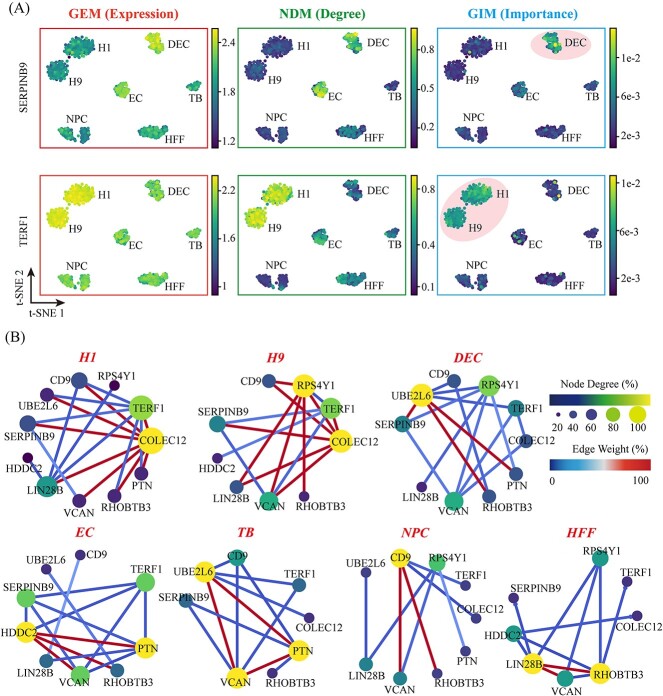
The comparative performance of GIM, NDM and GEM in identifying ‘Dark’ genes was evaluated on the *Chu-type* dataset. (**A**) The panels from left to right respectively depict the gene expression levels, network degree, and importance levels of SERPINB9 and TERF1. (**B**) The weighted correlation networks of ‘Dark’ genes and mark genes exhibit differential network sparsity across distinct cellular clusters.

Weighted gene correlation networks that encompass both ‘Dark’ and importance marker genes exhibit topological variations across seven cell clusters, as depicted in Figure 7B. The networks in highly efficient differentiated cells, including embryonic stem cells H1 and H9, are more intricate, featuring the key regulatory factor gene COLEC12, which encodes a C-type lectin family protein with a carbohydrate recognition domain. During cellular differentiation, the COLEC12 cell surface protein may play a role in intricate signal transduction processes. Notably, the gene COLEC12 is associated with dark genes such as SERPINB9 and TERF1, which may be critical for regulating cell apoptosis and the cell cycle during embryonic development [48–50]. Conversely, in cells with lower differentiation efficiency such as HFF, the gene correlation network is sparser, and the degree of correlation of the COLEC12 gene is considerably reduced, while its correlation with cryptic genes is disconnected.

## DISCUSSION AND CONCLUSION

Developing effective methods for accurately identifying single-cell heterogeneity is a fundamental challenge in scRNA-seq data analysis. Here, scGIR provides a method for constructing the weighted gene correlation networks at the single-cell level. The scGIR constructs a gene correlation network in an unsupervised manner and subsequently performs downstream analyses based on a relatively stable gene correlation network. This approach effectively addresses the issue of high noise levels commonly encountered in scRNA-seq analysis. However, solely relying on gene correlation networks analysis will lose information regarding gene expression representation to some extent. To address this limitation, we incorporated gene expression as correlation weights into the gene correlation network and determined gene importance indices in single cells by constructing a random walk model on the single-cell weighted gene correlation networks. scGIR transforms the single-cell GEM into a GIM, and the dimensionality reduction visualization of nine datasets shows that the GIM could significantly improve cell heterogeneity analysis accuracy and discover new cell subtypes.

The identification of key genes and regulatory factors, along with predicting their impact on gene regulatory networks using network-based approaches, is a crucial aspect of systems biology. Gene correlation networks represent intricate regulatory systems within cells that govern a multitude of physiological processes [18, 19, 51, 52]. Analyzing the topological structure and dynamic properties of gene correlation networks could reveal the stability, controllability, and robustness of cellular systems. Here, we introduce expression information to represent gene correlation weights based on the single-cell gene correlation network, further enhancing the network’s expressive power. The scGIR method transforms the GEM into an importance matrix, which has distinct advantages in separating cell subtypes, adapting to various clustering methods, and identifying ‘Dark’ genes when compared with GEM and NDM. scGIR exhibits stronger information extraction capabilities for sc-seq data.

In the regulation and determination of physiological processes, certain gene nodes positioned at the ‘hub’ of a gene network are of crucial importance [53–55]. ‘Hub’ genes, such as transcription factors, which may exhibit comparatively lower expression levels, have been evidenced to possess a heightened capability to distinguish cellular states [56]. Transcription factors, constituting approximately 8% of the human genome, are associated with various diseases and phenotypes [57]. Furthermore, internally matured microRNAs, characterized by their short length and low expression levels, play a crucial role in governing development, metabolism, and immune responses [58]. This class of key ‘hub’ genes is often overlooked when relying solely on single-cell gene expression matrices for analysis due to their low expression levels. However, through construction of single-cell weighted gene correlation networks and establishment of a random walk model, scGIR effectively identifies crucial ‘hub’ genes within the gene correlation network, potentially pivotal in cell fate determination. Illustrated in Figure 4B are these crucial ‘hub’ genes within seven cell clusters of the Chu-type dataset, pivotal in physiological processes like ubiquitination modification, DNA repair, and immune response. Among them, LIN28B, belonging to the lin-28 family, features a cold shock domain and CCHC zinc finger domains, involved in maintaining embryonic stem cell pluripotency and linked to let-7 microRNA expression suppression [59]. Utilizing scGIR for disease-related scRNA-seq datasets promises to significantly enhance intelligent drug target prediction and screening efficiency [60–62].

In a previous study, Dai *et al.*[17] developed a CSN method that established a way to infer single-cell gene correlation networks from single-cell sequencing data. However, the basic assumption of the CSN method for obtaining NDM is that the more correlation edges a gene has, the more important it is, which obviously ignores the status of the ‘neighbor genes’ in the entire gene correlation network. Here, we weighted the correlation edges by gene expression levels and systematically evaluated the importance of genes in the single-cell gene correlation network using a random walk model. Through the exploration of ‘Dark’ genes, we demonstrated the significant advantages of the scGIR. The nine scRNA-seq datasets selected in this study include multiple temporally differentiated datasets. scGIR not only identifies cell heterogeneity at different time points but also exhibits good temporal consistency. This suggests the applicability of scGIR in predicting the developmental potential of single cells.

Overall, the application of systems biology and complex network theory in the analysis of biological big data is becoming increasingly crucial, especially in identifying key genes and regulatory factors, as well as predicting their impact on cell fate decisions [63, 64]. The primary advantage of these approaches lies in their effectiveness in extracting intricate correlations from large-scale biological data, aiding in a better comprehension of the structure and functionality of gene regulatory networks. We identify inter-gene correlations from extensive scRNA-seq data, assessing the significance of each gene node in the network through a random walk model. Differential analysis of gene importance across distinct cell clusters allows for the identification of crucial genes and regulatory factors in the network. The scGIR algorithm provides a systematic perspective for understanding the complexity of gene regulatory networks. However, scGIR still faces limitations and challenges in dealing with single-cell weighted gene correlation networks. These networks are vast and dynamic, with significant differences even between networks originating from two cells within the same cluster. Uncovering the dynamic characteristics of complex single-cell weighted gene correlation networks will be crucial in elucidating the mechanisms behind cell fate decisions. Moreover, scGIR represents just an initial exploration of integrating complex network and systems biology theories into the development of algorithms for scRNA-seq data analysis, yet to comprehensively unveil the dynamic behaviors and regulatory strategies underlying single-cell weighted gene correlation networks. With advancements in sequencing technologies, the development of effective algorithms for analyzing single-cell multi-modal data and spatial transcriptomics data is imminent, where multi-layered complex networks and multi-scale dynamic modeling may serve as a crucial breakthrough.

**Key Points**

- The novel tool, scGIR, leverages single-cell weighted gene correlation network analysis to assess cellular heterogeneity, effectively addressing the issue of gene expression instability caused by transcriptional amplification noise and dropout events.
- scGIR algorithm performs random walks on the single-cell weighted gene correlation network to evaluate the importance of genes by statistically assessing the probability of gene node visitations.
- The dimensions of the gene importance matrix obtained from the scGIR algorithm align with those of the gene expression matrix, thereby avoiding an increase in the complexity of the analysis.
- scGIR algorithm offers a dual perspective at both the gene and single-cell network levels, excelling in identifying ‘Hub’ genes, ‘Dark’ genes, and network sparsity within various cell types, thereby offering an enhanced understanding of cellular heterogeneity.

## Supplementary Material

FigS1_bbae091

FigS2_bbae091

FigS3_bbae091

FigS4_bbae091

FigS5_bbae091

FigS6_bbae091

Supplementary_data_bbae091

## Data Availability

scGIR is available at https://github.com/XMU-Xu/scGIR.git.
