# Supplementary figures and images for: scGIR: deciphering cellular heterogeneity via gene ranking in single-cell weighted gene correlation networks
Source: Brief Bioinform. 2024 Mar 14;25(2):bbae091. doi: 10.1093/bib/bbae091 (PMC10940817; doi:10.1093/bib/bbae091)

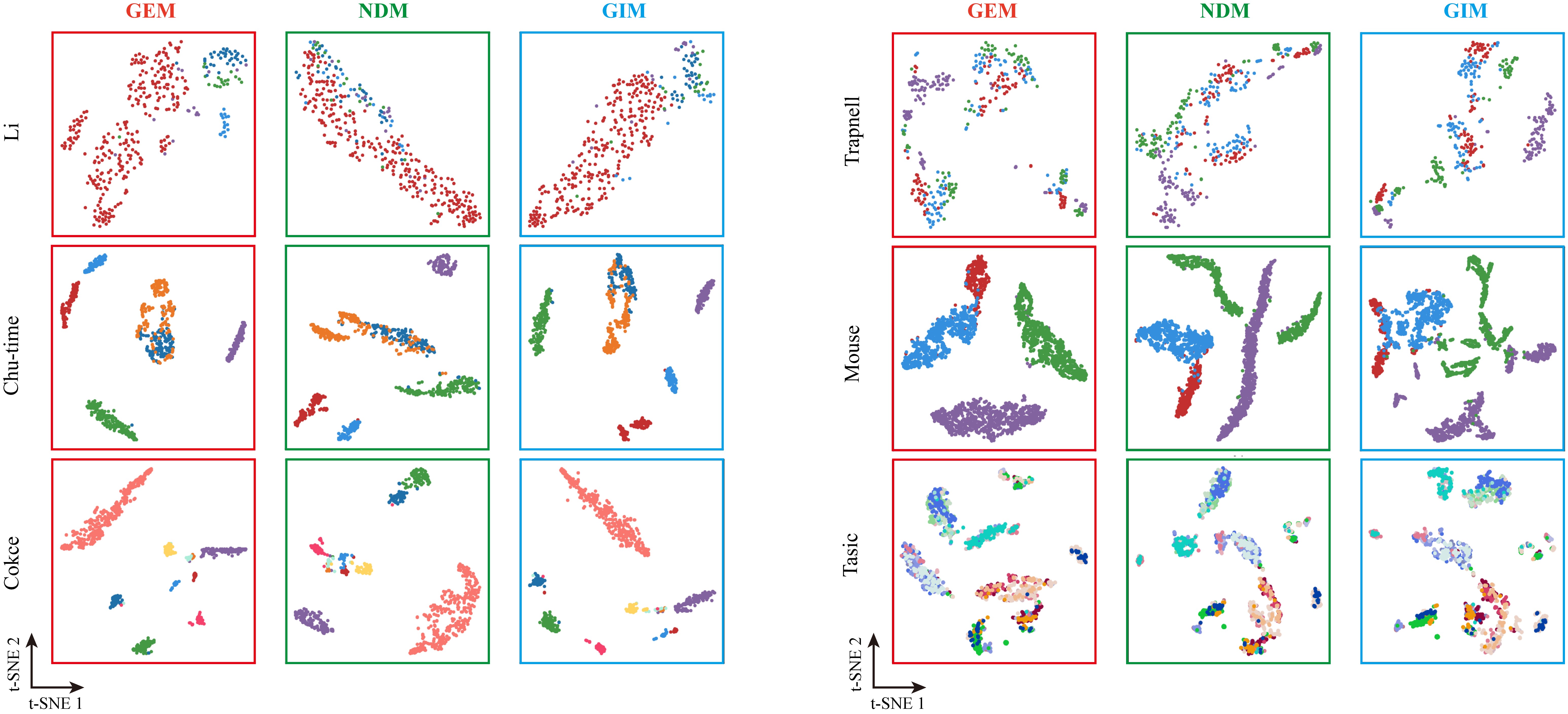

Supplement: FigS1_bbae091 [file figs1_bbae091.jpeg]

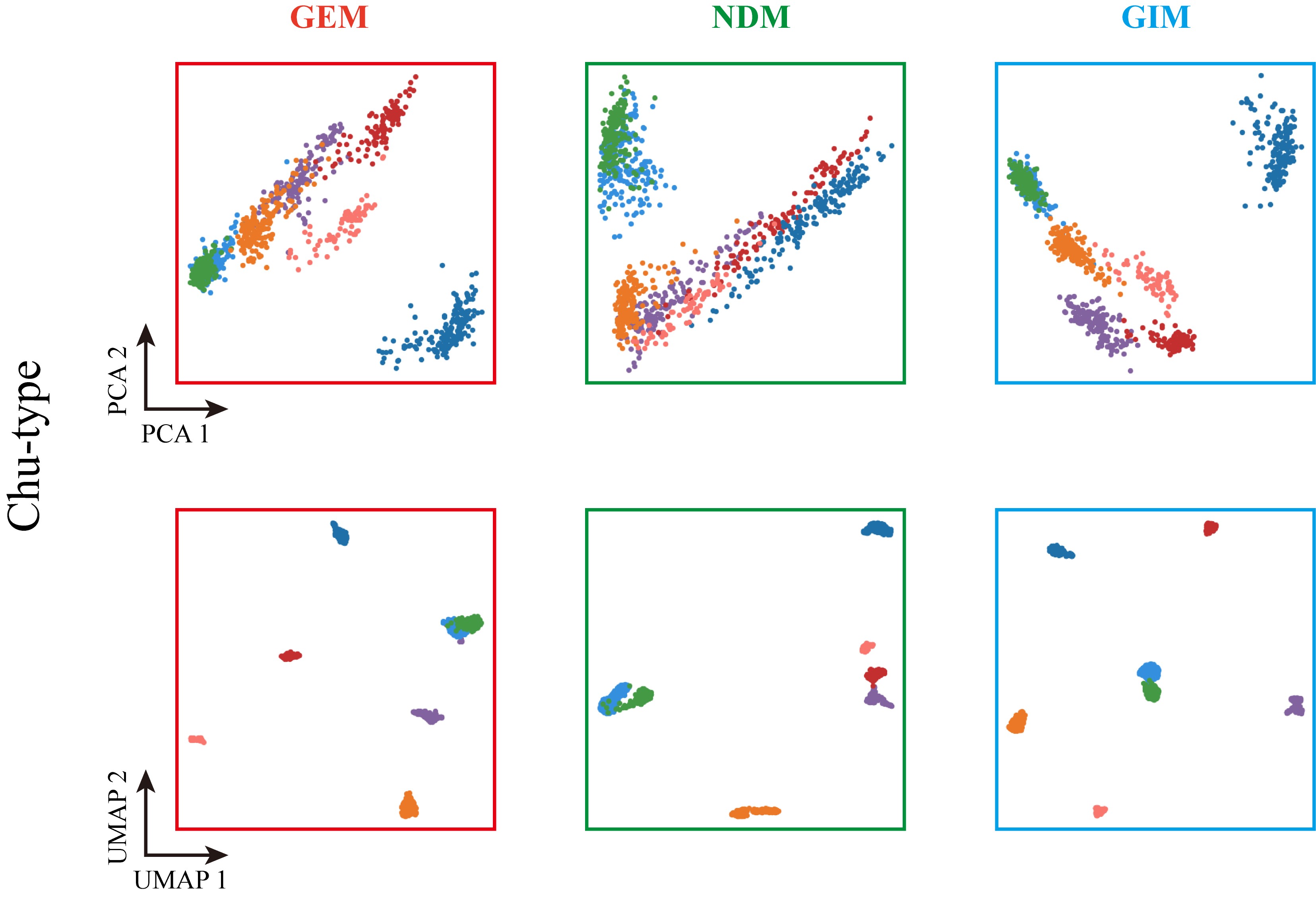

Supplement: FigS2_bbae091 [file figs2_bbae091.jpeg]

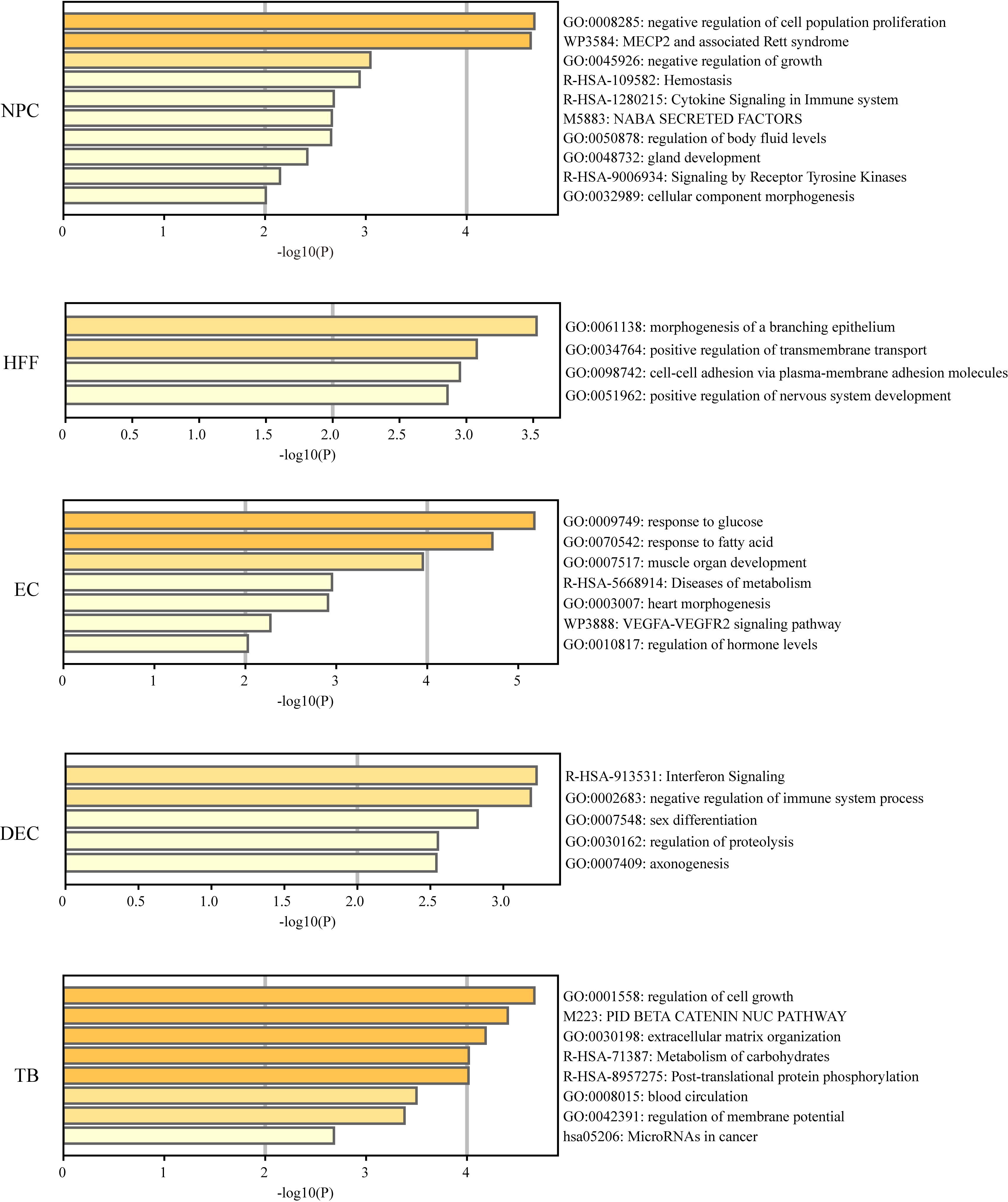

Supplement: FigS3_bbae091 [file figs3_bbae091.jpeg]

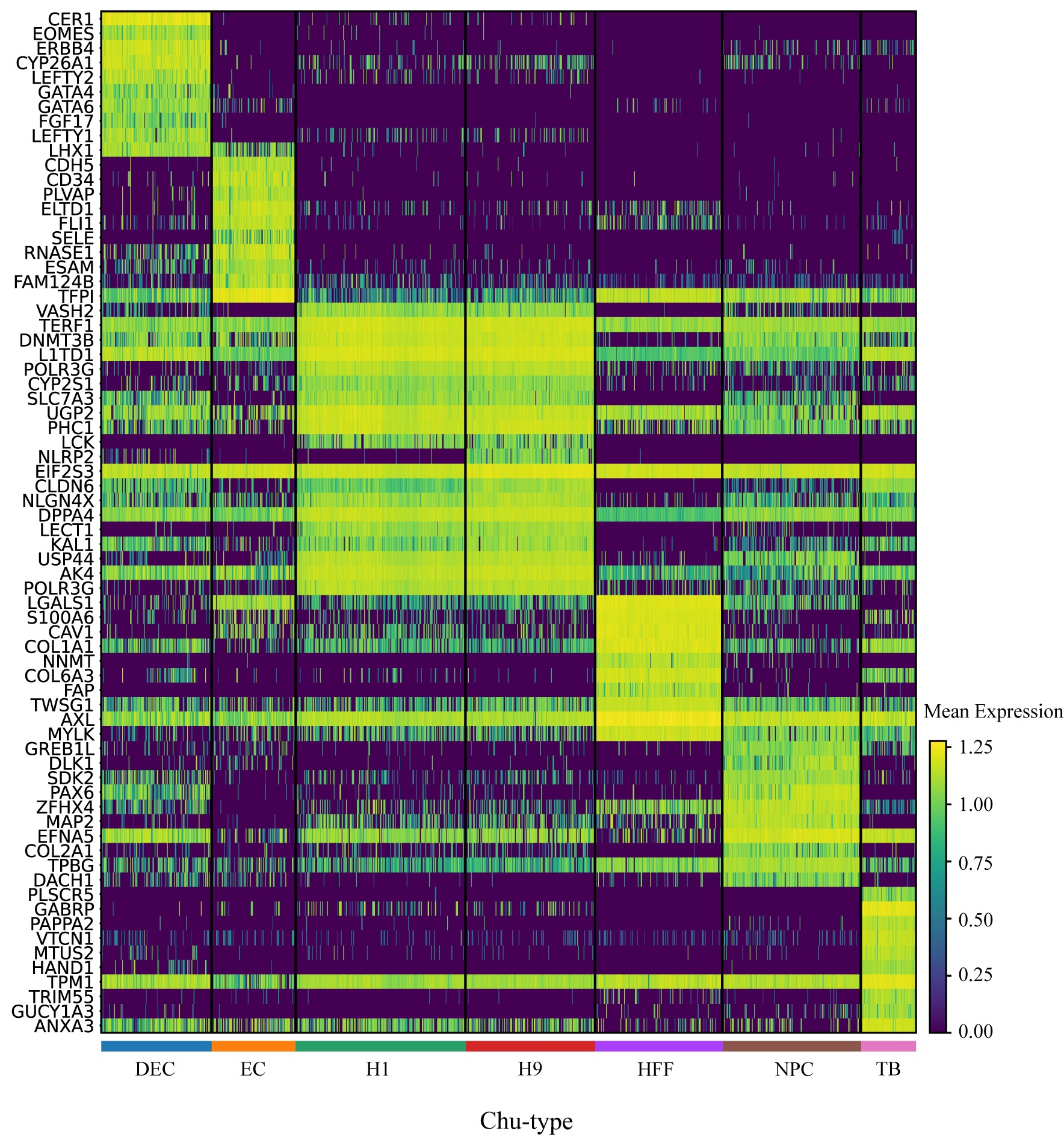

Supplement: FigS4_bbae091 [file figs4_bbae091.jpeg]

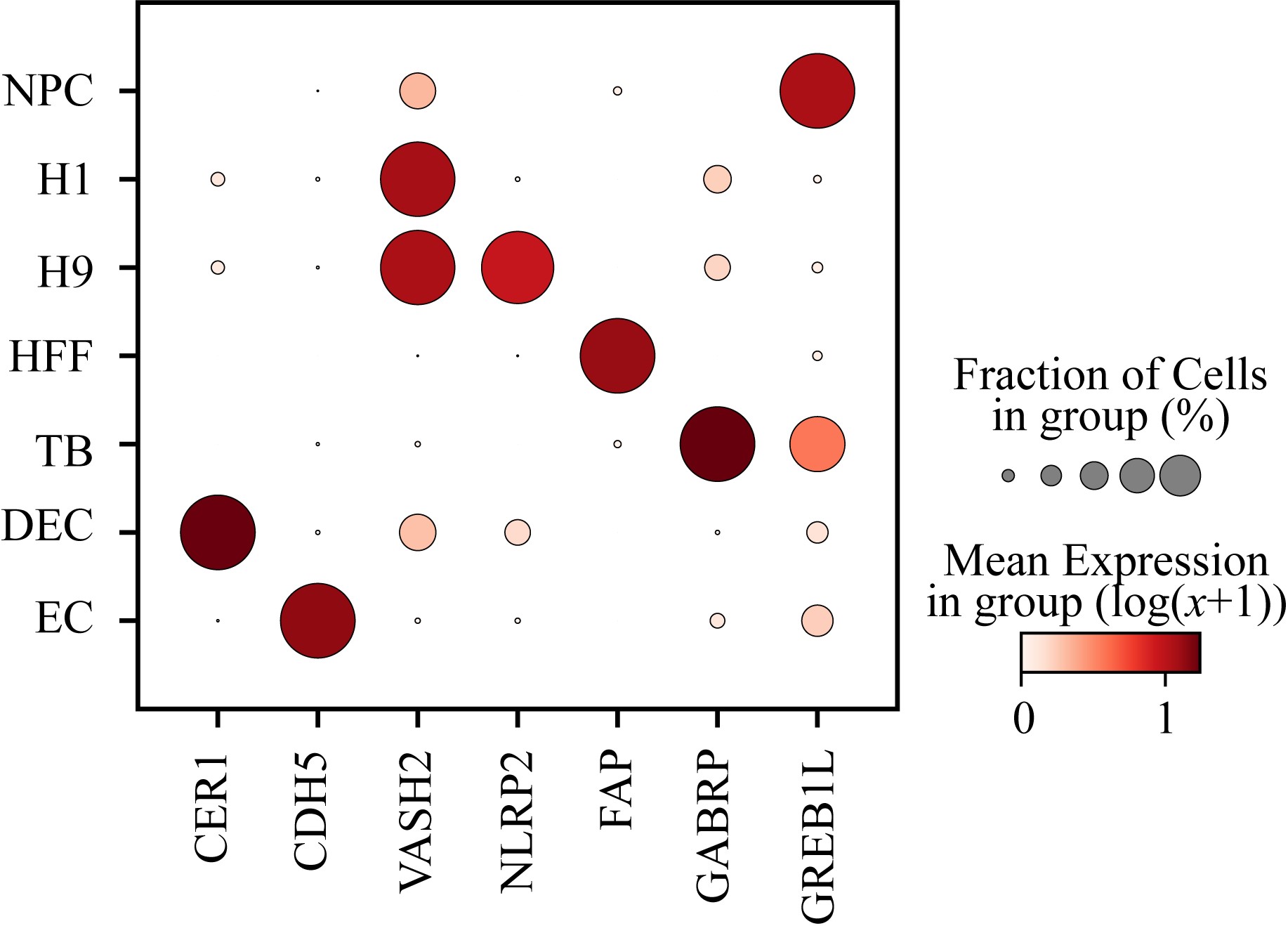

Supplement: FigS5_bbae091 [file figs5_bbae091.jpeg]

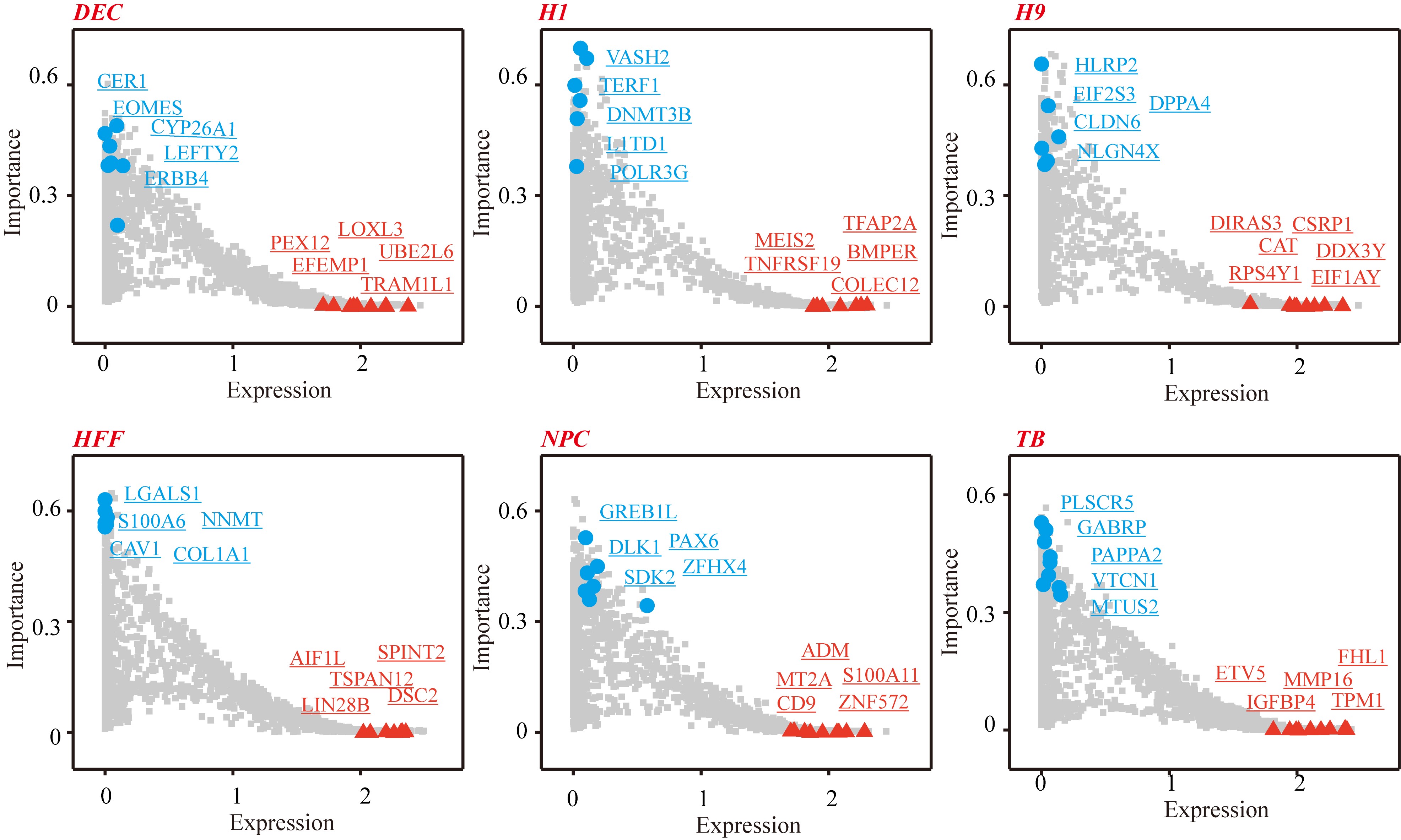

Supplement: FigS6_bbae091 [file figs6_bbae091.jpeg]
