# Supplementary material for: scGIR: deciphering cellular heterogeneity via gene ranking in single-cell weighted gene correlation networks
Source: Brief Bioinform. 2024 Mar 14;25(2):bbae091. doi: 10.1093/bib/bbae091 (PMC10940817; doi:10.1093/bib/bbae091)
Supplement: Supplementary_data_bbae091 [file supplementary_data_bbae091.docx]

**SUPPLEMENTARY INFORMATION
scGIR: deciphering cellular heterogeneity via gene ranking in single-cell weighted gene correlation networks**

**Fei Xu, Huan Hu, Hai Lin, Jun Lu, Feng Cheng, Jiqian Zhang, Xiang Li, and Jianwei Shuai**

Supplementary Figures 1-6

Figure S1. Comparison of GEM (red box), NDM (green box), and GIM (blue box) on non-linear dimensionality reduction (t-SNE) visualization for six other datasets.

Figure S2. PCA and UMAP for Dimensionality Reduction Visualization of Chu-type Datasets: GEM, NDM, and GIM.

Figure S3. Gene Ontology biological processes analysis of the mean importance marker genes in NPC, HFF, EC, DEC and TB cell clusters.

Figure S4. The heatmap shows the mean expression of the Top-10 marker genes obtained through the Wilcoxon rank-sum test for each cell cluster.

Figure S5. By employing quantitative expression analysis on the *Chu-type* dataset encompassing seven distinct cell clusters, the coloration of dots denotes the mean expression levels of marker genes within each cluster, while dot size illustrates the fraction of cells in the categories expressing a marker gene.

Figure S6. All genes in the DEC, H1, H9, HFF, NPC, NPC, and TB cell cluster mapped onto a two-dimensional space based on expression and importance, with marker genes highlighted separately.
